# Diversity of isoprene-degrading bacteria in phyllosphere and soil communities from a high isoprene-emitting environment: a Malaysian oil palm plantation

**DOI:** 10.1186/s40168-020-00860-7

**Published:** 2020-06-03

**Authors:** Ornella Carrión, Lisa Gibson, Dafydd M. O. Elias, Niall P. McNamara, Theo A. van Alen, Huub J. M. Op den Camp, Christina Vimala Supramaniam, Terry J. McGenity, J. Colin Murrell

**Affiliations:** 1 School of Environmental Sciences, University of East Anglia, Norwich Research Park, Norwich, NR4 7TJ UK; 2 UK Centre of Ecology & Hydrology, Lancaster Environment Centre, Library Avenue, Bailrigg, Lancaster, LA1 4AP UK; 3 Department of Microbiology, Faculty of Science, IWWR, Radboud University Nijmegen, Heyendaalseweg 135, NL-6525 AJ Nijmegen, The Netherlands; 4 School of Biosciences, Nottingham Centre of Sustainable Palm Oil, University of Nottingham-Malaysia, Jalan Broga, 43500 Semenyih, Selangor Darul Ehsan Malaysia; 5 School of Life Sciences, University of Essex, Colchester, UK

**Keywords:** Isoprene, Climate, Isoprene monooxygenase, DNA stable isotope probing, Oil palm, *isoA*

## Abstract

**Background:**

Isoprene is the most abundantly produced biogenic volatile organic compound (BVOC) on Earth, with annual global emissions almost equal to those of methane. Despite its importance in atmospheric chemistry and climate, little is known about the biological degradation of isoprene in the environment. The largest source of isoprene is terrestrial plants, and oil palms, the cultivation of which is expanding rapidly, are among the highest isoprene-producing trees.

**Results:**

DNA stable isotope probing (DNA-SIP) to study the microbial isoprene-degrading community associated with oil palm trees revealed novel genera of isoprene-utilising bacteria including *Novosphingobium*, *Pelomonas*, *Rhodoblastus*, *Sphingomonas* and *Zoogloea* in both oil palm soils and on leaves. Amplicon sequencing of *isoA* genes, which encode the α-subunit of the isoprene monooxygenase (IsoMO), a key enzyme in isoprene metabolism, confirmed that oil palm trees harbour a novel diversity of *isoA* sequences. In addition, metagenome-assembled genomes (MAGs) were reconstructed from oil palm soil and leaf metagenomes and putative isoprene degradation genes were identified. Analysis of unenriched metagenomes showed that *isoA*-containing bacteria are more abundant in soils than in the oil palm phyllosphere.

**Conclusion:**

This study greatly expands the known diversity of bacteria that can metabolise isoprene and contributes to a better understanding of the biological degradation of this important but neglected climate-active gas.

Video abstract.

## Background

Isoprene (2-methyl-1,3-butadiene), with atmospheric emissions of 400–600 Tg year^−1^, is the most abundantly produced biogenic volatile compound (BVOC) on Earth. This is approximately one third of the total volatile organic compound (VOC) emissions and almost equal to global annual emissions of methane [1, 2]. Due to its volatile nature and high reactivity, isoprene plays a complex role in atmospheric chemistry and hence, climate. In pristine environments with low levels of nitrogen oxides (NOx), isoprene reacts with hydroxyl radicals (OH) and reduces the oxidising capacity of the atmosphere, which in turn increases the lifetime of greenhouse gases such as methane. However, when nitric oxide (NO) is present at high levels, as commonly found in urban areas, reactions with isoprene form nitrogen dioxide (NO_2_) and increase the trophospheric levels of ozone, which has detrimental impacts on air quality and human health and impedes progress towards many of the UN sustainable development goals [3–6]. Conversely, the products of isoprene oxidation can form secondary aerosols and act as cloud condensation nuclei, resulting in a global cooling effect [7].

While there are some industrial sources of isoprene (0.8 Tg year^−1^), primarily from the production of synthetic rubber [8], the vast majority of isoprene emissions (~ 90%) originate from terrestrial plants [9, 10], with small contributions from marine algae (0.1–12 Tg year^−1^), bacteria, fungi and animals [9, 11–16]. The enzyme responsible for isoprene production in plants is isoprene synthase, the presence and activity of which can vary significantly even between closely related species [17–20]. In isoprene-emitting plants, isoprene is produced in the chloroplast via the methyl-erythritol 4-phosphate (MEP) pathway [21]. Isoprene synthase is responsible for converting dimethylallyl diphosphate (DMAPP) to isoprene. Despite the fact that up to 2% of the carbon fixed by isoprene-emitting plants contributes to the synthesis of isoprene [22, 23], its role in plants is not fully understood. It has been reported that isoprene improves the resilience of plants to oxidative, thermal and biotic stresses [23–26]. However, the molecular mechanisms behind these processes have not yet been fully elucidated. In addition, it has been recently suggested that isoprene may play a role in regulating gene expression in plants [27].

While the production and atmospheric fate of isoprene has been well studied, biological consumption in the isoprene biogeochemical cycle remains relatively unexplored. Field chamber and continuous-flow studies have shown that soils are a biological sink for isoprene at environmentally relevant concentrations [28–30]. Several bacterial strains capable of growing on isoprene as a sole carbon and energy source have been isolated from soils, phyllosphere and aquatic environments (reviewed in [31]). Most of these strains are Gram-positive Actinobacteria, although more recent studies have led to the isolation of Gram-negative Proteobacteria expanding the known diversity of isoprene-degrading bacteria [32]. All characterised isoprene-utilising microorganisms contain six genes (*isoABCDEF*) that encode the isoprene monooxygenase (IsoMO) enzyme, which catalyses the first step in the isoprene degradation pathway. Adjacent genes *isoGHIJ* encode enzymes involved in the subsequent steps of isoprene metabolism [33]. The IsoMO belongs to the soluble diiron monooxygenase (SDIMO) family [34], and the α-subunit contains the diiron centre at the putative active site. The gene encoding this IsoMO α-subunit, *isoA*, is highly conserved in isoprene-utilising bacteria and is an excellent marker gene for isoprene degraders [35, 36]. The development of probes targeting *isoA* has been a successful approach to investigate the distribution, diversity and abundance of isoprene degraders in several environments, including oil palm soils and leaves [35]. However, it is important to combine the use of *isoA* probes with other cultivation-independent techniques such as DNA stable isotope probing (DNA-SIP) [37] to investigate the diversity of active isoprene degraders in the environment and to better assess the role that microbes play in the biogeochemical cycle of isoprene. Indeed, previous DNA-SIP experiments with ^13^C-labelled isoprene have led to the identification of novel genera of isoprene degraders in phyllosphere and soil environments, such as *Sphingopyxis*, *Ramlibacter* and *Variovorax* [32, 38]. In turn, the sequencing information provided by these DNA-SIP experiments has allowed the design of targeted cultivation strategies that resulted in the isolation of representative strains of these novel genera of isoprene degraders [32], which now can be used as model microorganisms to study how isoprene metabolism is regulated.

The oil palm tree (*Elaeis guineensis*) is one of the highest isoprene-producing trees, with estimated emissions of 175 μg g^−1^ (dry leaves) h^−1^ [39]. Oil palm is a major crop across Southeast Asia and is the source of 30% of the world’s vegetable oil [40], and in countries such as Malaysia, it covers > 85% of total agricultural land, with an ongoing annual land usage increase of 6.9% attributed solely to oil palm cultivation [40, 41]. Therefore, the vast expansion of a single crop that emits such high amounts of isoprene has raised serious concerns about the impact of oil palm plantations on air quality [42]. Here, we combine cultivation-dependent techniques with DNA-SIP, *isoA* and 16S rRNA gene amplicon sequencing, and focussed metagenomics, to study the isoprene-degrading microbial communities associated with oil palm trees in a Malaysian plantation, both from the phyllosphere and from the soil nearby.

## Results and discussion

### Identification of active isoprene-degrading bacteria using DNA-SIP

#### Diversity of bacteria from soils in the vicinity of oil palm trees

Analysis of 16S rRNA gene amplicon sequences showed that the unenriched bacterial community from soils in the vicinity of oil palm trees was very similar across replicates, confirming that extraction and handling procedures were consistent (Fig S1 and Fig S2 show the relative abundance (RA) of 16S rRNA genes in these environmental samples). The unenriched soil microbial community (S T0) was mainly composed of Proteobacteria (40.8 ± 0.5% RA), Actinobacteria (13.1 ± 0.7%), Bacteroidetes (11.2 ± 1.4%) and Acidobacteria (10.8 ± 0.6%, Fig S1), all of which are dominant phyla in soils [43–46]. The most abundant genera were *Rhodoplanes* (5.9 ± 0.1%) and *Flavobacterium* (4.0 ± 0.9%; Fig S2).

**Fig. 1:**
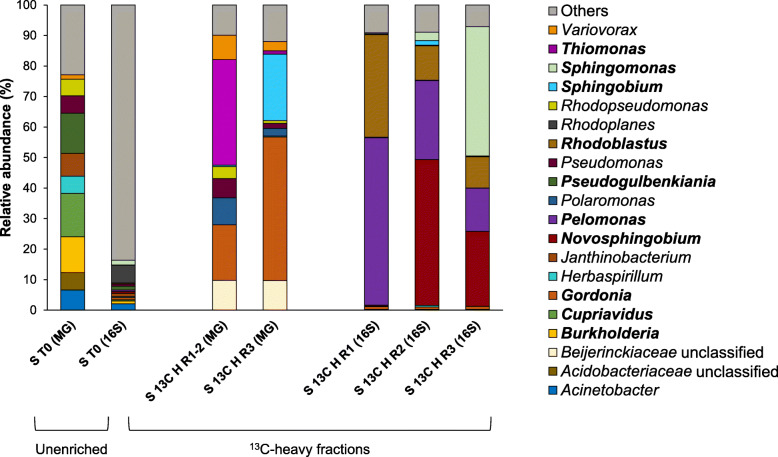
Bacterial community profile of oil palm soil samples. Bacterial diversity of the unenriched (T0) and labelled (heavy; H) fractions of ^13^C-isoprene soil incubations was analysed by 16S rRNA gene amplicon (16S) and metagenomics (MG) sequencing. The unenriched bacterial community (S T0) represents the average of three biological replicates. ^13^C-heavy DNA from replicates 1 and 2 were combined before MG sequencing due to their highly similar 16S rRNA gene community profile shown by DGGE, resulting in sample S 13C H R1-2 (see the “Methods” section). Only genera with > 5% RA in at least one of the conditions are represented. Genera present at > 10% in any sample are shown in bold. Genera with < 5% RA are grouped in “others”. For complete 16S rRNA gene amplicon sequencing data, including individual replicates and ^12^C-isoprene controls, see Fig S2

Soils were then enriched with ^13^C-isoprene to identify the active isoprene degraders in this environment through DNA-SIP (see the “Methods” section). Sequencing of the 16S rRNA genes of the ^13^C-heavy fractions showed that, although there was considerable inter-sample variability, *Rhodoblastus* (10.2–33.7%) and *Pelomonas* (14.2–54.9%) were highly enriched in all soil replicates (S 13C H; Fig. 1). *Novosphingobium* was one of the major genera labelled in the ^13^C-heavy fractions of replicates 2 (S 13C H R2) and 3 (S 13C H R3) representing 47.8% and 24.5%, respectively. Finally, *Sphingomonas* dominated the isoprene-degrading community of replicate 3 (S 13C H R3) with a RA of 42.4% (Fig. 1). These four genera had 19- to 90-fold higher RA in ^13^C-heavy (S 13C H) than in the ^13^C-light (S 13C L) soil fractions and constituted 28.7–40.2% of the total microbial community of the unfractionated soils incubated with ^13^C-isoprene (13C UF; Fig S2), which strongly suggest that they are active isoprene degraders. As expected, *Novosphingobium*, *Pelomonas*, *Sphingomonas* and *Rhodoblastus* also dominated the ^12^C-isoprene-incubated microbial community (S 12C L), and each genus had a very similar RA to those of the unfractionated ^13^C-samples (S 13C UF; Fig S2).

Previous DNA-SIP experiments and cultivation-dependent studies have identified members of *Sphingomonadaceae* (*Sphingopyxis*) and *Comamonadaceae* (*Ramlibacter* and *Variovorax*) as isoprene degraders with a functional IsoMO [32, 36, 38]. However, this is the first evidence that other genera of these families such as *Sphingomonas*, *Novosphingobium* and *Pelomonas* are likely to be able to metabolise isoprene. In addition, *Rhodoblastus* is the first member of the *Beijerinckiaceae* family to be implicated in isoprene degradation. Therefore, it will be interesting to attempt to isolate representative strains of this genus in future studies in order to confirm this ability.

DNA-SIP and 16S rRNA gene amplicon sequencing showed that the oil palm soil harbours a distinct isoprene-degrading bacterial community from soils beneath temperate trees that emit high levels of isoprene, such as willow. DNA-SIP experiments using willow soil incubated with ^13^C-labelled isoprene identified *Rhodococcus*, *Ramlibacter* and *Variovorax* as the major genera labelled in the ^13^C-heavy fractions [32]. However, these genera represented < 1% of the ^13^C-heavy (S 13C H) fractions from oil palm soil. Also, other well-characterised isoprene-degrading microorganisms, such as *Gordonia*, *Nocardioides*, *Mycobacterium* and *Sphingopyxis* species [32, 47, 48], constituted only a small part of the isoprene-degrading community (< 1%) from soils taken from the vicinity of oil palm trees.

Both unenriched samples and heavy DNA fractions from these soil incubations were also subjected to metagenomic sequencing. Community composition of raw reads was assessed with MetaPhlAn2 [49]. As MetaPhlAn2 uses a range of clade-specific marker genes to assess the phylogeny of the metagenomics reads, results differed slightly from those obtained using 16S rRNA gene amplicon sequencing analysis. According to the phylogenetic analysis of the soil metagenomes, the unenriched soil (S T0) community was dominated by Proteobacteria (81.5%), Actinobacteria (7.8%) and Acidobacteria (6.3%; Fig S1), confirming the results obtained by the analysis of the 16S rRNA gene amplicon sequencing data. The most abundant bacteria in the unenriched soils that could be classified at the genus level belonged to *Cupriavidus* (14.2%), followed by *Pseudogulbenkiania* (13.2%) and *Burkholderia* (11.7%; Fig. 1).

Metagenomic sequencing revealed that ^13^C-heavy fractions from replicates 1 and 2 (S 13C H R1-2) were dominated by *Thiomonas* (34.5%) and *Gordonia* (18.2%), whereas replicate 3 (S 13C H R3) had a higher abundance of *Gordonia* (47%) and *Sphingobium* (21.8%; Fig. 1). However, these genera represented < 1% of the ^13^C-heavy fractions in the 16S rRNA gene amplicon sequencing data. It is not surprising to find members of *Gordonia* dominating the isoprene-degrading community, since strains from this genus have been shown to contain a complete isoprene degradation gene cluster [48]. However, this study provides the first evidence that *Thiomonas* and *Sphingobium* species may be also able to catabolise isoprene.

#### Diversity of bacteria from the phyllosphere of oil palm trees

**Fig. 2:**
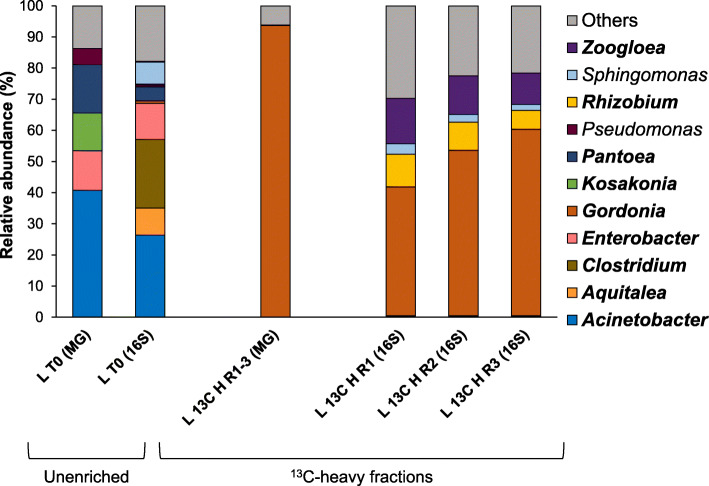
Bacterial community composition of oil palm phyllosphere samples. Bacterial diversity profile of unenriched (T0) and labelled (heavy; H) fractions of ^13^C-isoprene incubations of oil palm leaf samples was analysed by 16S rRNA gene amplicon (16S) and metagenomics (MG) sequencing. The unenriched bacterial community (L T0) represents the average of three biological replicates. ^13^C-heavy DNA from replicates 1, 2 and 3 of leaf incubations were combined before MG sequencing due to their highly similar 16S rRNA gene community profile shown by DGGE, resulting in sample L 13C H R1-3 (see the “Methods” section). Only genera with > 5 RA% in at least one of the conditions are represented. Genera present at > 10% in any sample are shown in bold. Genera with < 5% RA are grouped in “others”. For complete 16S rRNA gene amplicon sequencing data, including individual replicates and ^12^C-isoprene controls, see Fig S3

The bacterial community of unenriched oil palm leaf (L T0) samples was dominated by Proteobacteria (74.5 ± 0.3%, Fig S1), which is not surprising since Proteobacteria have been found to be the most abundant phylum in the phyllosphere of several plant species [50–53]. Firmicutes also constituted a major component of the unenriched bacterial community from oil palm leaves (22.1 ± 0.2%, Fig S1), as has been reported for some trees and agricultural plants [51, 52, 54, 55]. The most abundant genera in the oil palm phyllosphere were *Acinetobacter* (26.4 ± 0.7%), followed by *Clostridium* (22.0 ± 0.2%) and *Enterobacter* (11.6 ± 0.2%; Fig 2).

The 16S rRNA gene amplicon sequencing data showed that the diversity of the isoprene-degrading community of the samples incubated with ^13^C-isoprene (L 13C H) was highly consistent between replicates, with *Gordonia* (51.4 ± 9.4%) and *Zoogloea* (12.3 ± 2.2%) being the most abundant genera (Fig. 2). The RA of *Gordonia* and *Zoogloea* was 84.9 and 58.2-fold higher in the ^13^C-heavy (L 13C H) compared to the ^13^C-light (L 13C L) fraction, respectively (Fig S3), indicating that they are active isoprene degraders. In addition, these two genera constituted 10.8% of the total microbial community of the ^13^C-unfractionated (L 13C UF) samples and, as expected, were also highly abundant in the ^12^C-isoprene-incubated (L 12C L) microbial community (13.6%; Fig S3).

Strains of *Gordonia* that grow on isoprene as sole carbon and energy source have been isolated previously from leaves of an oil palm tree in the Palm House of Kew Gardens, London [32]. However, although a number of SIP experiments with ^13^C-isoprene have been performed with samples from a wide range of environments, including the phyllosphere, estuaries and soils, no members of the *Zoogloea* genus or the order *Rhodocyclales* have been identified as active isoprene degraders [32, 36, 38, 48]. Here, the identification of *Zoogloea* as an isoprene degrader indicates that the variety of microorganisms able to metabolise this important climate-active gas is greater than previously known.

*Rhizobium* also had a relatively high RA (8.5 ± 2.2%) in all replicates from ^13^C-heavy fractions (L 13C H) compared to the unenriched (L T0) samples (Fig. 2). However, its RA was 2.2-fold higher in the ^13^C-heavy (L 13C H) than the ^13^C-light (L 13C L) fractions, which is the same ratio observed between the ^12^C-heavy (L 12C H) and the ^12^C-light (L 12C L) fractions (Fig S3). Therefore, based on these data, and since no strains of this genus have been isolated from this environment to corroborate its ability to degrade isoprene, *Rhizobium* spp. cannot be yet confirmed as isoprene degraders.

Previous SIP experiments exploring the phyllosphere of other high isoprene-emitting trees from temperate regions, such as poplar, identified *Rhodococcus* and *Variovorax* as the major players in isoprene degradation [38]. However, in our experiment, the phyllosphere from tropical oil palm trees yielded a distinct profile of active isoprene degraders, with *Gordonia* and *Zoogloea* being the main genera enriched in the ^13^C-heavy fractions and *Rhodococcus* and *Variovorax* showing a low RA (2.1 ± 0.4% and < 1%, respectively). The RA of other well-characterised isoprene degraders such as *Sphingopyxis*, *Ramlibacter*, *Nocardioides* or *Mycobacterium* [32, 48] also represented < 1% of the labelled bacterial community from oil palm leaves (L 13C H).

Phylogenetic analysis of the unenriched leaf (L T0) metagenomes confirmed that the unenriched bacterial community of the oil palm phyllosphere was overwhelmingly dominated by Proteobacteria (99.1% RA; Fig S1). At the genus level, metagenomics analysis also supported the 16S rRNA gene amplicon sequencing data, since *Acinetobacter* (40.8% RA) and *Enterobacter* (12.7% RA) were highly abundant in the unenriched phyllosphere community, together with *Pantoea* (15.5% RA; Fig. 2).

Metagenomic data showed that *Gordonia* constituted 93.7% of the isoprene-degrading community of oil palm leaves (L 13C H R1-3; Fig. 2), in accordance with the 16S rRNA gene amplicon sequencing results. However, no *Zoogloea* sequences were identified in the ^13^C-heavy fractions in the metagenomic analysis probably due to the different approach that MetaPhlAn2 uses to assign the phylogeny of the reads compared to the 16S rRNA gene amplicon analysis.

#### Comparison of isoprene degraders from soils and phyllosphere of oil palm trees

Studying the microbial diversity associated with plants is an essential step to understand host-microbiome interactions. However, only a few studies comparing microbial communities of phyllosphere and soils associated with the same plant species have been conducted to date (e.g. [54, 56, 57]). Here, we show that the unenriched bacterial communities from oil palm soils (S T0) and leaves (L T0) are distinct even at the phylum level (Fig S1), as reported for other plant species [54], although both soil and leaves are dominated by Proteobacteria. The 16S rRNA gene amplicon sequencing data also revealed that the active isoprene-degrading bacteria from soil samples were phylogenetically more diverse than those from the oil palm phyllosphere (see above).

When comparing the unenriched soil (S T0) and leaf (L T0) communities, it is interesting to note that although each major player in isoprene degradation was present in these contrasting environments at similar RA (Table S1), they responded differently to isoprene enrichment (S 13C H and L 13C H). This suggests that the physiochemical conditions and/or interactions with other groups of microorganisms shape the composition of the isoprene-degrading community in a particular environment.

In addition, unenriched soil and leaf oil palm metagenomes (S T0 and L T0) were analysed for the presence and relative abundance of *isoA* genes. Metagenomic data showed that *isoA*-containing bacteria were 5-fold more abundant in soil samples (1% of bacteria) than in the phyllosphere samples (0.2% of bacteria). Metagenomes obtained in previous studies of unenriched samples from high isoprene-emitting trees from temperate regions, such as poplar [38] and willow [32], were also analysed for comparison. Results showed that 0.7% of bacteria from soil beneath a willow tree and 0.02% of bacteria from poplar leaves contained *isoA* genes. These data, though sparse, showed the same trend observed in the oil palm environment, with soils containing greater numbers of bacteria with the genetic potential to degrade isoprene than the phyllosphere. This finding is surprising considering the greater availability of isoprene in the canopy than at ground-level [58] and indicates that soils could be a more important sink for isoprene than previously thought.

### Recovery of metagenome-assembled genomes

Assembled contigs from soil and leaf metagenomes were used to reconstruct metagenome-assembled genomes (MAGs) using MaxBin2 [59]. A total of 20 MAGs from soil and 52 from leaf samples were obtained (Table S2). From these, two MAGs from soils and three from leaf ^13^C-heavy DNA metagenomes with > 75% completeness and < 10% contamination contained genes encoding homologous polypeptides to IsoABCDEF (*E* < 1e−40). MAGs containing IsoMO-encoding genes from soil incubations were taxonomically classified as *Novosphingobium* and *Rhizobiales*, and leaf MAGs were classified as *Gordonia*, *Zoogloeaceae* and *Ralstonia* (Table S3).

**Fig. 3:**
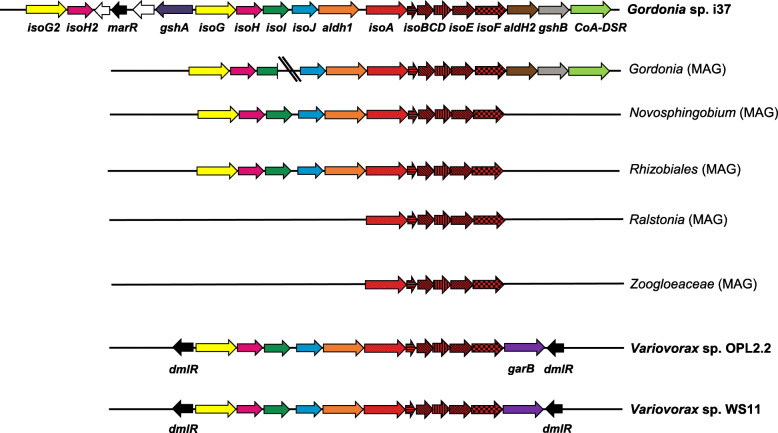
Isoprene metabolic gene clusters from representative isoprene-degrading strains (in bold) and metagenome-assembled genomes (MAGs). Genes encoding IsoMO (*isoABCDEF*) are coloured in red. Adjacent genes not involved in isoprene degradation are coloured in white. Regulatory genes are shown in black. “\\” represents a discontinuity between two DNA contigs. *Variovorax* sp. OPL2.2 was isolated in this study from oil palm leaf enrichments.

**Fig. 4:**
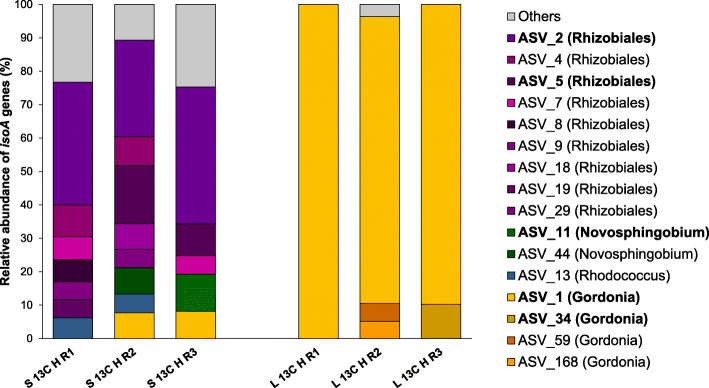
Relative abundance and diversity of *isoA* genes in ^13^C-heavy fractions from oil palm incubations. *isoA* sequences in ^13^C-heavy fractions from oil palm soil (S 13C H) and leaf (L 13C H) samples were analysed by *isoA* amplicon sequencing (see the “Methods” section). Only AVSs with > 5% RA in at least one replicate are represented. ASVs present at > 10% in any sample are shown in bold. ASVs closely related to IsoA from *Rhizobiales* MAG are represented in purple; ASVs with highest homology to IsoA from *Novosphingobium* MAG are shown in green; ASVs closely related to IsoA from *Rhodococcus* are coloured in blue; ASVs with highest homology to IsoA from *Gordonia* MAG are represented in orange. ASVs with RA < 5% are grouped as “others”. For complete *isoA* gene amplicon sequencing data, see Table S5

The *Novosphingobium* soil-associated MAG contained the full isoprene degradation gene cluster (*isoABCDEFGHIJ*) on a single contig along with *aldH1*, which encodes an aldehyde dehydrogenase [33]. However, no further accessory genes were recovered (Fig. 3). The products of these genes shared 76.2–100% amino acid identity (Table S4) with the corresponding polypeptides from *Sphingopyxis* sp. OPL5, a *Sphingomonadales* strain isolated from oil palm [32]. When the diversity and abundance of *isoA* genes in the ^13^C-heavy DNA fractions from soil samples were analysed by *isoA* amplicon sequencing, two amplicon sequence variants (ASVs), ASV44 and ASV11, were identified, closely related to the IsoA from the *Novosphingobium* MAG (> 99% amino acid identity; Table S5). These two ASVs represented 7.9% of the *isoA* genes of the ^13^C-heavy DNA fraction from replicate 2 and 11% from replicate 3, respectively (Fig. 4).

A *Rhizobiales* MAG was also reconstructed from ^13^C-heavy DNA soil samples. Although this MAG had high completeness (97.6%) and low contamination (2.5%), it showed a high strain heterogeneity (79%; Table S3), indicating that the MAG originated from DNA from one or more closely related microorganisms, thus making identification at a higher resolution difficult. Despite this, a complete isoprene degradation gene cluster (*isoABCDEFGHIJ*) plus *aldH1* were located in a single contig (Fig. 3). When these genes were translated, they exhibited an amino acid identity of 54.8–84.9% to the homologous proteins from *Sphingopyxis* sp. OPL5, except for *isoD*, the product of which was more closely related to IsoD from *Ramlibacter* sp. WS9 [32] (Table S4). *isoA* gene amplicon sequencing analysis revealed that the ASVs closely related to the IsoA from the *Rhizobiales* MAG (> 71% amino acid identity; Table S5) dominated the *isoA*-containing bacterial community from all ^13^C-heavy DNA soil replicates, comprising a total of 64.9 ± 7.7% of the *isoA* genes in these samples (Fig. 4).

The *Gordonia* phyllosphere MAG was identified to the species level as *Gordonia polyisoprenivorans* i37 [48] (average nucleotide identity; ANI: 96.7%) and contained all IsoMO-encoding genes *isoABCDEF*, along with upstream genes *isoGHIJ* (Fig. 3). This MAG also contained *aldH2*, *CoA-DSR* and *gshB*, which are accessory genes often found within the isoprene degradation gene cluster in Gram-positive bacteria and encode an aldehyde dehydrogenase, a CoA-disulfide reductase and a glutathione synthetase, respectively [33]. All the genes associated with the isoprene degradation pathway recovered in the *Gordonia* MAG encoded polypeptides that shared > 94% amino acid identity to the corresponding proteins from *Gordonia polyisoprenivorans* i37, except for IsoB (82.1%) and AldH1 (83%; Table S4). When the ^13^C-heavy DNA fractions from leaf samples were analysed by *isoA* amplicon sequencing, several ASVs with a high percentage of amino acid identity to the IsoA from the *Gordonia* MAG (> 93%; Table S5) were recovered. However, ASV1, which showed 100% amino acid identity to IsoA from the *Gordonia* MAG, overwhelmingly dominated the *isoA*-containing bacterial community, representing 91.9 ± 7.3% of the *isoA* genes in these samples (Fig. 4).

The remaining two phyllosphere MAGs, *Zoogloeaceae* and *Ralstonia*, contained genes that encoded homologous polypeptides to IsoABCDEF, although they showed a low amino acid identity to the corresponding IsoMO proteins from well-characterised isoprene degraders (34.1–52.5%; Table S4). In addition, no gene homologues to *isoGHIJ* were recovered from these MAGs (Fig. 3). While the absence of homologues to *isoGHIJ* and the low sequence identity to IsoABCDEF indicates that these bacteria may harbour a novel isoprene degradation pathway, especially considering that these MAGs were recovered from ^13^C-heavy DNA fractions metagenomes, we cannot be absolutely certain that they are from bona fide isoprene degraders. These *isoA*-like sequences were not identified by the *isoA* amplicon analysis, suggesting that the relatively high number of mismatches with the *isoA* primers prevented successful PCR amplification. Therefore, further targeted isolations of these bacteria and/or expression of these *isoABCDEF* genes in a heterologous host [38] are required to establish that these are genuine isoprene degraders.

Finally, if *Zoogloeaceae* and *Ralstonia* microorganisms could be confirmed as bona fide isoprene degraders, the abundance of bacteria with the potential to metabolise isoprene would increase from 1 to 2% in oil palm soil and from 0.1 to 0.2% in phyllosphere unenriched samples. Similarly, the RA of *isoA*-containing bacteria would also increase in both willow soil (from 0.7 to 1.3% of bacteria) and poplar leaf (from 0.02 to 0.05% of bacteria) unenriched samples, indicating that isoprene degraders could be more abundant in the environment than previously thought. However, more samples from contrasting ecosystems need to be explored to support this hypothesis.

### Isolation and characterisation of *Variovorax* sp. OPL2.2

Cultures set up using material from the DNA-SIP experiments from soils and leaves were subcultured three times at 2-week intervals with 25 ppmv isoprene before plating onto minimal medium with isoprene as sole carbon source (see the “Methods” section). A strain belonging to the genus *Variovorax* isolated from leaf enrichments was able to grow on isoprene as sole carbon and energy source (Fig S4).

Although DNA-SIP experiments have shown that *Variovorax* plays an important role in isoprene degradation in the phyllosphere [38], *Variovorax* sp. OPL2.2 is the first strain of this genus isolated from a tropical environment. When genomic DNA of OPL2.2 was screened for *isoA*, it yielded a PCR product which had a translated sequence with 99.4% amino acid identity to IsoA from *Variovorax* sp. WS11, an isoprene degrader isolated from willow soil [32].

The genome of this new isolate from oil palm, *Variovorax* sp. OPL2.2, was sequenced using Illumina and Nanopore technologies to confirm that it contained a full isoprene degradation gene cluster. Assembly of both Illumina and Nanopore reads and downstream analysis with CheckM [60] revealed that the *Variovorax* sp. OPL2.2 genome assembly comprised of 50 contigs totalling 8.5 Mbp and had a 98.4% completeness, 1.1% contamination and a GC content of 67.4%. Finally, after automatic annotation by Prokka [61], 8200 predicted coding sequences were found in the *Variovorax* sp. OPL2.2 genome.

Genome analysis confirmed that *Variovorax* sp. OPL2.2 contained *isoABCDEF* encoding IsoMO. *isoGHIJ* and *aldH1* genes, which are involved in the subsequent steps of isoprene metabolism, were located upstream *isoABCDEF* in an identical layout to those of many bona fide isoprene-degrading strains [38] (Fig. 3). *garB*, which encodes a glutathione disulfide reductase, was also located in the same gene cluster (Fig. 3). *isoABCDEFGHIJ*, *aldH1* and *garB* encoded polypeptides with high amino acid identity (99.7–100%) to those from *Variovorax* sp. WS11. Indeed, ANI analysis (> 99.9%) revealed that *Variovorax* sp. OPL2.2 is the same species as *Variovorax* sp. WS11.

## Conclusions

The area of agricultural land dedicated to cultivating oil palm has increased dramatically over the last 40 years and continues to increase annually due to palm oil demand from the food industry, domestic products and biofuel production [42]. Growth of a high isoprene-emitting crop and the consequent reactions of isoprene with NOx have raised concerns about the impact of large oil palm plantations on air quality and the regional delivery of the UN sustainable development goals. Therefore, to better understand the role that microbes play in mitigating the effects of this climate-active gas, it is essential to examine the microbial diversity and abundance of isoprene-degrading bacteria. In this study, we show that oil palm harbours a unique and distinct community of isoprene degraders compared to other high isoprene-emitting trees from temperate environments and contains higher numbers of bacteria with the genetic potential to metabolise this climate-active gas, especially soils beneath oil palm trees. The isoprene-utilising bacterial community from oil palm soils was phylogenetically more diverse than that of the oil palm phyllosphere, with *Novosphingobium, Pelomonas, Rhodoblastus* and *Sphingomonas* being found in soils and *Gordonia* and *Zoogloea* on oil palm leaves. Analysis of MAGs revealed that bins from these genera contained isoprene degradation gene clusters, and amplicon sequencing data showed that oil palm trees contain novel *isoA* sequences, many of which were highly similar to *isoA* genes recovered from *Novosphingobium*, *Gordonia* or *Rhizobiales* MAGs. The discovery of novel isoprene-degrading bacteria enhances a more robust *isoA* database necessary to utilise the *isoA* probes and assess the distribution, abundance and activity of the isoprene degraders in the environment.

## Methods

### DNA-stable isotope probing

Three trees from different locations in an oil palm plantation in Palong (Negeri Sembilan, Malaysia) were sampled on 12 November 2018 (Table S6). Soil (50 g) was collected at a depth of 0–5 cm within the palm circle of three 28-year old oil palm trees after removing vegetation from the surface of the soil. Five healthy leaflets from lower canopy fronds of the corresponding trees were sampled to allow comparison of the diversity of isoprene-degrading bacteria between the oil palm phyllosphere and adjacent soils. Soils and leaf samples were sent to the laboratory of Prof. Niall McNamara at the UK Centre for Ecology and Hydrology in Lancaster, where appropriate facilities to import soils into the UK were available, for processing. Cells were extracted from the soils as follows: 5 g of soils were resuspended in 50 ml sterile distilled water in a sterile 250-ml conical flask. Flasks were then shaken at 150 rpm for 30 min at room temperature to dislodge cells from soil particles. Soil suspensions were decanted into a 50-ml measuring cylinder and left undisturbed for 1 h to allow sedimentation of soil particles. The aqueous layer was decanted into a sterile flask. This treatment was repeated including a sonication step for 5 min in a water bath (Mettler Electronics) to optimise recovery of cells that were attached to soil particles. Soils particles were appropriately disposed of by autoclaving, and the aqueous layers of both treatments were then combined and transported in sealed vials to the University of East Anglia, where microcosms with soil washings were set up.

Soil DNA-SIP enrichments were set up in triplicate and consisted of 80 ml of combined soil washings in 2-l air-tight bottles containing 25 ppmv of either ^12^C or ^13^C-labelled isoprene. Soil microcosms were incubated at 30 °C in the dark with agitation at 150 rpm. Five leaves per oil palm tree (the same trees used for the soil microcosms) were cut into approximately 10 cm length and 5 cm width and placed in sterile glass bottles containing 250 ml sterile distilled water. Leaf samples were sonicated for 5 min in a water bath (Mettler Electronics) and agitated at 150 rpm at room temperature for 1 h to dislodge microbial cells from plant material. Leaf washings were then filtered through 0.22 μm cellulose nitrate membrane filters (Pall) to concentrate cells. Filters were washed with 40 ml of Ewers minimal medium [62]. Subsequently, filters were discarded, and the washings transferred to 2-l air-tight bottles to form the basis of the leaf microcosms. Phyllosphere microcosms were then amended with 25 ppmv of either ^12^C- or ^13^C-labelled isoprene. Leaf DNA-SIP enrichments were set up in triplicate and incubated at 30 °C in the dark with shaking (150 rpm). Consumption of isoprene by soil and leaf microcosms was monitored with a Fast Isoprene Sensor (Hills-Scientific) and replenished when the headspace concentration fell below 10 ppmv.

Sampling of soil and leaf DNA-SIP incubations was performed as follows: 10 ml aliquots were collected at T0 and after 5 days (12.5 μmol C assimilated g^−1^) of incubation for soil microcosms. Ten milliliters aliquots from microcosms set up with leaf washings were collected at T0 and after 10 days (50 μmol C assimilated g^−1^) of incubation. Soil and leaf aliquots were then spun down, and supernatants were discarded. After that, pellets were resuspended with a 1-ml solution containing sodium phosphate and MT buffers included in the FastDNA Spin Kit for Soil (MP Biomedicals) and transferred to Lysing matrix E tubes to proceed with the DNA extraction according to the manufacturer’s instructions. 0.5 to 2 μg DNA per sample was separated into heavy (^13^C-labelled) and light (^12^C-unlabelled) DNA by isopycnic ultracentrifugation as previously described [36]. DNA in each fraction was quantified using a Qubit dsDNA HS Assay kit (ThermoFisher Scientific) following the manufacturer’s instructions. The density of each fraction was estimated by refractometry using a Reichert AR200 refractometer (Reichert Analytical Instruments). Heavy and light DNA fractions from each sample were identified by plotting DNA abundance vs fraction density (Fig S5) and used for subsequent downstream analysis.

### 16S rRNA gene amplicon sequencing

To investigate the bacterial diversity in samples from DNA-SIP experiments, 16S rRNA genes of DNA extracted from unenriched (T0), unfractionated (UF), labelled (heavy; H) and unlabelled (light; L) soil and leaf fractions were amplified with bacterial primers 341F and 785R [63]. Duplicate PCRs for each sample were pooled before purification of PCR amplicons with a High Pure PCR product purification kit (Roche) according to the manufacturer’s instructions. It was not possible to obtain amplicons from ^12^C-heavy DNA (S 12C H) samples arising from SIP experiments with soils due to the low amount of DNA present in these fractions (below the detection limit of 0.2 ng of the Qubit dsDNA HS Assay kit). DNA libraries from purified 16S rRNA gene amplicons were prepared and sequenced at MrDNA (Shallowater, TX, USA) with Illumina MiSeq technology, obtaining an average of 100,757 16S rRNA reads per sample with an average length of 300 bp. Sequence data were processed using MrDNA analysis pipeline. Briefly, reads were first joined and depleted of barcodes. Then, short sequences (< 150 bp) and sequences with ambiguous base calls were removed. Resultant sequences were denoised, and operational taxonomic units (OTUs) were defined with clustering at 97% similarity, followed by removal of singleton sequences and chimeras. Taxonomy of OTUs was then assigned using BLASTn (http://blast.ncbi.nlm.nih.gov) against a curated database derived from RPDII (http://rdp.cme.msu.edu) and NCBI (www.ncbi.nlm.nih.gov).

### *isoA* gene amplicon sequencing

*isoA* genes of DNA extracted from ^13^C-heavy soil and leaf fractions were amplified with primers isoA14F and isoA511R [35]. Duplicate PCRs for each replicate enriched with ^13^C-isoprene were pooled before purification of PCR amplicons with a High Pure PCR product purification kit (Roche) as above. DNA libraries from purified PCR products were prepared and sequenced at MrDNA (Shallowater, TX, USA) using Illumina MiSeq technology, obtaining an average of 13,542 reads per sample with an average length of 300 bp.

*isoA* amplicon sequencing data were analysed with the Bioconductor package DADA2 (version 1.6) [64] after demultiplexing the reads and removing primer sequences. Reads were then trimmed to 275 nucleotides and quality-filtered if their expected error was greater than two. Sequences were then denoised using the estimated error rates, and resultant reads were dereplicated. Subsequently, forward and reverse reads were merged, chimeric sequences were discarded and the DADA2 algorithm was used to infer individual amplicon sequence variants (ASVs). ASVs were then manually checked by BLASTx [65]. Those ASVs with hits not related to IsoA were discarded, obtaining a final set of 28 ASVs for soil and 6 ASVs for leaf enrichments.

### Metagenomic analysis of oil palm soil and leaf DNA-SIP samples

The 16S rRNA gene profile of each biological replicate of unenriched (T0), labelled (heavy; H) and unlabelled (light; L) fractions from leaf and soil samples was analysed by denaturing gradient gel electrophoresis (DGGE, see below). As they showed highly similar profiles, biological replicates from both unenriched soils (S T0 R1, S T0 R2 and S T0 R3) and phyllosphere (L T0 R1, L T0 R2 and L T0 R3) samples and ^13^C-heavy fractions from leaves (L 13C H R1, L 13C H R2 and L 13C H R3) were combined in equal proportions for metagenomics sequencing (Fig S6), resulting in samples S T0, L T0 and L 13C H R1-3, respectively. However, the community profile of ^13^C-heavy fraction from soils replicate 3 (S 13C H R3) showed some differences compared to those from ^13^C-heavy fractions from replicates 1 and 2 (S 13C H R1 and S 13C H R2; Fig S6). Therefore, ^13^C-heavy DNA soil fractions from replicates 1 and 2 were pooled (S 13C H R1-2) for downstream analysis, whereas ^13^C-heavy DNA from replicate 3 (S 13C H R3) was sequenced separately. Libraries were prepared by MrDNA (Shallowater, TX, USA), resulting in an average insert size of 700 bp for soils T0 (S T0), 710 bp for leaves T0 (L T0), 643 bp for soils ^13^C-heavy fractions from replicates 1 and 2 (S 13C H R1-2), 622 bp for soils ^13^C-heavy fraction replicate 3 (S 13C H R3) and 639 bp for ^13^C-heavy leaves fractions (L 13C H R1-3). Libraries were then pooled in equimolar ratios of 1 nM and sequenced as paired ends using the Illumina NovaSeq 6000 system.

Metagenomic reads were quality-filtered using the iu-filter-quality-minoche script [66] included in Illumina-utils (version 1.4.4) [67], obtaining an average of 19,000,000 quality-filtered reads per sample with an average length of 143 bp. Taxonomy of unassembled metagenomes was analysed using MetaPhlAn2 (version 2.0) [49].

The abundance of *isoA* genes in unassembled oil palm (this study), willow [32] and poplar [38] unenriched metagenomes was determined by tBLASTn of IsoA sequences from ratified isoprene-degrading bacteria against the raw reads (*E* ≤ 1e−4). Each potential IsoA sequence retrieved from the analysis of metagenomes was manually checked by BLASTx against a database of IsoA proteins from bona fide isoprene degraders and discarded if they showed < 50% amino acid identity. Only unique hits were counted. Hit numbers were normalized against read number of the smallest metagenome, to the smallest gene length and to hits of *recA*. Hits of *recA* in unenriched metagenomes were determined by tBLASTn (*E* ≤ 1e−6) of RecA sequences from a database obtained from RDP’s FunGene [68]. Quality-filtered reads from soil and leaf samples were assembled using metaSPAdes (version 3.13) [69] with kmers 21, 33 and 55, and the quality of each assembly was analysed with MetaQUAST (version 4.6.3) [70]. N50 values were ~ 1 kb for all metagenome assemblies except for the unenriched soil (S T0) sample, which had an N50 value of 684. Complete statistics of metagenome assemblies are shown in Table S2.

Assembled contigs were used to reconstruct metagenome-assembled genomes (MAGs) using MaxBin2 (version 2.2.2) [59]. MAGs completeness and contamination were assessed and taxonomically assigned using CheckM (version 1.0.18) [60]. A total of 20 MAGs from soil and 52 from leaf samples were obtained. Those MAGs with > 75% completeness and < 5% contamination were then reassembled and taxonomically verified using the “reassemble_bins” and “classify_bins” modules of the metaWRAP pipeline (version 1.2.2) [71] to improve assembly and increase the likelihood of obtaining full isoprene degradation gene clusters. Local BLAST databases were constructed and screened for the presence of homologues to known isoprene degradation proteins IsoABCDEFGHIJ and other polypeptides associated with the pathway such as AldH1, GshB and GarB using a cut-off value of *E* < 1e−10 in permissive searches and *E* < 1e−40 in restrictive searches. A total of two MAGs from soils and three from leaves were identified to be of interest based on a completeness of > 75%, contamination < 10% and the presence of IsoABCDEF homologues (Table S3). Finally, MAGs were annotated using Prokka (version 1.13.3) [61].

### Isolation and characterisation of isoprene-degrading bacteria

Once soils and leaf microcosms set up for DNA-SIP experiments had assimilated 50 μmol C per gram of sample (10 days for leaves and 11 days for soils), they were diluted 1/10 into sealed 120-ml serum vials containing 10 ml Ewers minimal medium [62] to isolate isoprene-degrading strains from these environments. Vials were supplemented with 25 ppmv isoprene and incubated at 30 °C with shaking (150 rpm). Vials were subcultured three times at 2-week intervals before plating onto Ewers medium agar. These plates were incubated in air-tight jars containing isoprene vapour (1%, v/v) as sole carbon source. After 4 days, colonies with different morphologies were inoculated in 120-ml serum vials containing 10 ml Ewers medium and 25 ppmv isoprene to determine isoprene consumption of isolates in liquid medium. Consumption of isoprene was monitored using a gas chromatograph fitted with a flame ionization detector as previously described [33]. Those cultures that consistently consumed isoprene were checked for purity by plating onto rich medium R2A agar (Oxoid) and by phase-contrast microscopy (Zeiss Axioscop).

Growth curves of selected isolates on isoprene were performed as follows: bacterial strains were first grown on Ewers medium with glucose (10 mM) for 48 h at 30 °C. Cultures were then pelleted and washed three times with Ewers medium with no carbon sources and adjusted to an OD_600_ of 0.8. Cell suspensions were then inoculated into 120-ml serum vials containing 20 ml of fresh Ewers medium amended with isoprene vapour (5%, v/v) as sole carbon source. Cultures were incubated at 30 °C, and growth was estimated by measuring cell density at OD_600_ with a UV-1800 spectrophotometer (Shimadzu).

Genomic DNA of isoprene-degrading strains was extracted using a Wizard Genomic DNA purification kit (Promega) according to the manufacturer’s guidelines. The phylogeny of isolates was identified by PCR amplification of their 16S rRNA genes with primers 27F and 1492R [72] and subsequent Sanger sequencing (Eurofins genomics). Genomic DNA was also examined for the presence of *isoA* genes using PCR primers isoA14F and isoA511R, which are specific for the detection of *isoA*, encoding the IsoMO α-subunit [35]. PCR conditions for amplification of *isoA* genes consisted of an initial step of 94 °C for 2 min, followed by 31 cycles of 95 °C for 15 s, 54 °C for 30 s, 72 °C for 1 min and a final extension step of 72 °C for 7 min as described in [35].

### Illumina and Nanopore sequencing of genomic DNA from *Variovorax* sp. OPL2.2

Genomic DNA from *Variovorax* sp. OPL2.2 for Illumina and Nanopore sequencing was extracted using a phenol/chloroform/isoamyl alcohol method described by Wilson [73]. For Illumina sequencing, library preparation was done using the Nextera XT kit (Illumina) according to the manufacturer’s instructions. The library was checked for quality and size distribution using the Agilent 2100 Bioanalyzer (Agilent) and the Qubit dsDNA HS Assay Kit (Thermo Fisher Scientific). The library was then denatured and sequenced with Illumina Miseq technology (San Diego, CA, USA). Paired-end sequencing of 2 × 300 bp was performed using the MiSeq Reagent Kit v3 (San Diego, CA, USA) according to the manufacturer’s protocol. Illumina sequencing of *Variovorax* sp. OPL2.2 genomic DNA resulted in 1,274,620 reads.

Genomic DNA (0.9 μg) from *Variovorax* sp. OPL2.2 was used to prepare the library for Nanopore sequencing. Library construction was performed using the Ligation Sequencing Kit 1D (SQK-LSK109) in combination with the Native barcoding Expansion Kit (EXP-NBD104) according to the manufacturer’s protocol (Oxford Nanopore Technologies). DNA fragments were repaired and A-tailed using the NEBNext® FFPE DNA Repair Mix and NEBNext® Ultra™ II End Repair/dA-Tailing Module (New England Biolabs). After purification with AMPure XP beads (Beckman Coulter Life Sciences), selected barcodes were ligated using the Blunt/TA Ligase Master Mix (New England Biolabs). After ligation of the barcodes and bead clean-up, the library was quantified using the Qubit dsDNA HS Assay Kit (Thermo Fisher Scientific). Thereafter, adapters were ligated using the NEBNext® Quick Ligation Module (New England Biolabs). The library was then purified and quantified with the Qubit dsDNA HS Assay Kit (Thermo Fisher Scientific) and sequenced using a Flow Cell (R9.4.1) and MinION device (Oxford Nanopore Technologies) according to the manufacturer’s instructions.

Nanopore sequencing resulted in 44,530 reads (N50 = 10.5 kb). Base-calling and de-multiplexing after sequencing were done using the Guppy Base-calling Software (Oxford Nanopore Technologies, Limited Version 3.2.4+d9ed22f) resulting in 13,966 reads, selecting for a minimal sequence length of 3,000 bp.

Assembly of both Illumina and Nanopore reads to obtain a full-length genome was done using Unicycler [74]. Assembly of *Variovorax* sp. OPL2.2 reads yielded 50 contigs with a total length of 8,516,444 bp, and the largest contig being 2,344,322 bp. Completeness of the assembled genome was analysed by CheckM [60] resulting in 98.4% completeness and 1.1% contamination. Automatic annotation of *Variovorax* sp. OPL2.2 genome was performed using Prokka [61].

Finally, average nucleotide identity (ANI) between *Variovorax* sp. OPL2.2 and *Variovorax* sp. WS11 [32, 38] was calculated using the online ANI calculator available on the Kostas Lab website (http://enve-omics.ce.gatech.edu/ani/index, accessed 10/12/2019).

### Denaturing gel gradient electrophoresis

Bacterial 16S rRNA genes from DNA samples arising from DNA-SIP experiments were amplified using primers 341F-GC [75] and 907R [76]. To visualise 16S rRNA gene profiles of the bacterial communities from soil and leaf incubations, denaturing gel gradient electrophoresis (DGGE) was performed following the protocol described by El Khawand et al. [36].

## Supplementary information

**Additional file 1: Fig S1.** Bacterial diversity profile of the oil palm unenriched (T0) community at the phyla level. **Fig S2.** Bacterial community profiles of oil palm soil samples analysed by 16S rRNA gene amplicon sequencing. **Fig S3.** Bacterial community composition of oil palm leaf samples analysed by 16S rRNA gene amplicon sequencing. **Fig S4.** Growth curve of *Variovorax* sp. OPL2.2 on isoprene as sole carbon and energy source. **Fig S5.** DNA retrieved as function of density of each fraction recovered after isopycnic ultracentrifugation. **Fig S6.** 16S rRNA gene profiles of oil palm soil and phyllosphere samples analysed by DGGE. **Table S1.** Relative abundance of key isoprene-degrading bacterial genera in oil palm soil and leaf samples. **Table S2.** Statistics for metagenome assemblies. **Table S3.** Metagenome-assembled genomes (MAGs) that contain genes encoding proteins homologous to IsoABCDEF (E < 1e-40). **Table S4.** MAGs genes encoding polypeptides homologous to proteins involved in isoprene metabolism from ratified isoprene-degrading strains. **Table S5.** ASVs retrieved from *isoA* amplicon sequencing analysis of ^13^C-heavy DNA from soil and leaf incubations. **Table S6.** Location of oil palm trees used to set up soil and leaf DNA-SIP incubations.

## Data Availability

Amplicon sequencing and metagenomic data generated in this study were deposited to the sequence read archives (SRA) under Bioproject PRJNA272922 (Biosamples SAMN14771267 - SAMN14771280).
