# Supplementary material for: Diversity of isoprene-degrading bacteria in phyllosphere and soil communities from a high isoprene-emitting environment: a Malaysian oil palm plantation
Source: Microbiome. 2020 Jun 3;8:81. doi: 10.1186/s40168-020-00860-7 (PMC7271495; doi:10.1186/s40168-020-00860-7)
Supplement: Supplementary file 2 — Additional file 1: Fig S1. Bacterial diversity profile of the oil palm unenriched (T0) community at the phyla level. Fig S2. Bacterial community profiles of oil palm soil samples analysed by 16S rRNA gene amplicon sequencing. Fig S3. Bacterial community composition of oil palm leaf samples analysed by 16S rRNA gene amplicon sequencing. Fig S4. Growth curve of Variovorax sp. OPL2.2 on isoprene as sole carbon and energy source. Fig S5. DNA retrieved as function of density of each fraction recovered after isopycnic ultracentrifugation. Fig S6. 16S rRNA gene profiles of oil palm soil and phyllosphere samples analysed by DGGE. Table S1. Relative abundance of key isoprene-degrading bacterial genera in oil palm soil and leaf samples. Table S2. Statistics for metagenome assemblies. Table S3. Metagenome-assembled genomes (MAGs) that contain genes encoding proteins homologous to IsoABCDEF (E < 1e-40). Table S4. MAGs genes encoding polypeptides homologous to proteins involved in isoprene metabolism from ratified isoprene-degrading strains. Table S5. ASVs retrieved from isoA amplicon sequencing analysis of 13C-heavy DNA from soil and leaf incubations. Table S6. Location of oil palm trees used to set up soil and leaf DNA-SIP incubations. [file 40168_2020_860_MOESM1_ESM.docx]

**Additional File 1**

**Diversity of isoprene-degrading bacteria in phyllosphere and soil communities from a high isoprene-emitting environment: a Malaysian oil palm plantation**

Ornella Carrión^1ǂ*^, Lisa Gibson^1ǂ^, Dafydd M.O. Elias^2^, Niall P. McNamara^2^, Theo A. van Alen^3^, Huub J.M. Op den Camp^3^, Christina Vimala Supramaniam^4^, Terry J. McGenity^5^, J. Colin Murrell^1*^

^1^School of Environmental Sciences, University of East Anglia, Norwich Research Park, Norwich, NR4 7TJ, UK

^2^Centre of Ecology and Hydrology, Lancaster University, Bailrigg, Lancaster, LA1 4AP, UK

^3^Department of Microbiology, Faculty of Science, IWWR, Radboud University Nijmegen, Heyendaalseweg 135, NL-6525 AJ Nijmegen, The Netherlands

^4^School of Biosciences, Nottingham Centre of Sustainable Palm Oil, University of Nottingham-Malaysia, Jalan Broga, 43500 Semenyih, Selangor Darul Ehsan, Malaysia

^5^School of Life Sciences, University of Essex, Colchester, UK

^ǂ^These authors contributed equally to this work

*Corresponding authors:

J Colin Murrell, School of Environmental Sciences, University of East Anglia

Norwich Research Park, NR4 7TJ, UK

E-mail: j.c.murrell@uea.ac.uk

Tel: (+44) 01603 592959

Ornella Carrión, School of Environmental Sciences, University of East Anglia

Norwich Research Park, NR4 7TJ, UK

E-mail: o.carrion-fonseca@uea.ac.uk

Tel: (+44) 01603 592239


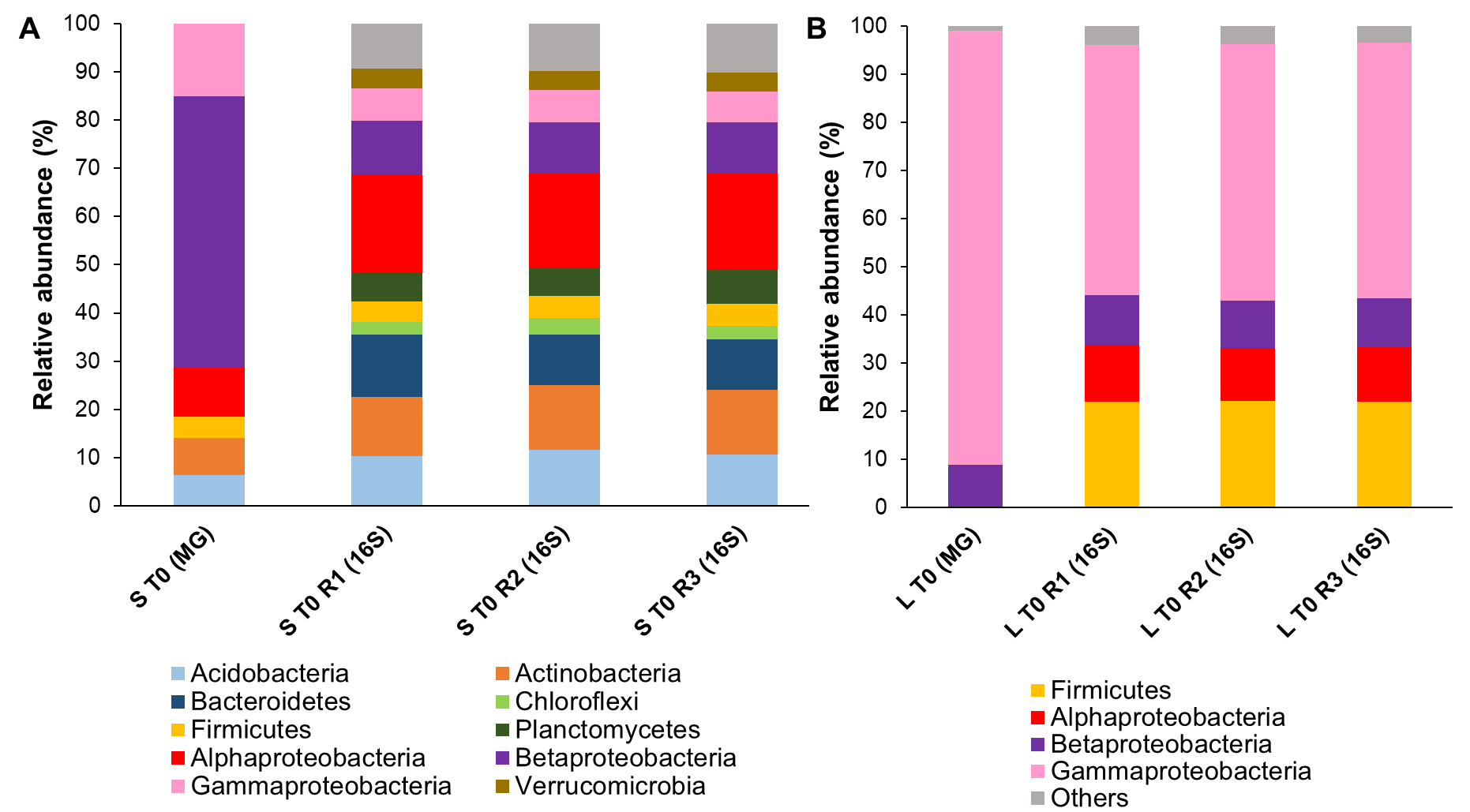


**Fig S1. Bacterial diversity profile of the oil palm unenriched (T0) community at the phyla level.** Bacterial community composition was analysed by 16S rRNA gene amplicon (16S) and metagenomics (MG) sequencing. A: soil T0 samples; B: phyllosphere T0 samples. Only phyla with >2% relative abundance in at least one of the samples are represented. The Proteobacteria phylum is subdivided into Alpha-, Beta- and Gammaproteobacteria classes. Bacterial phyla with <2% relative abundance are recorded as “Others”.


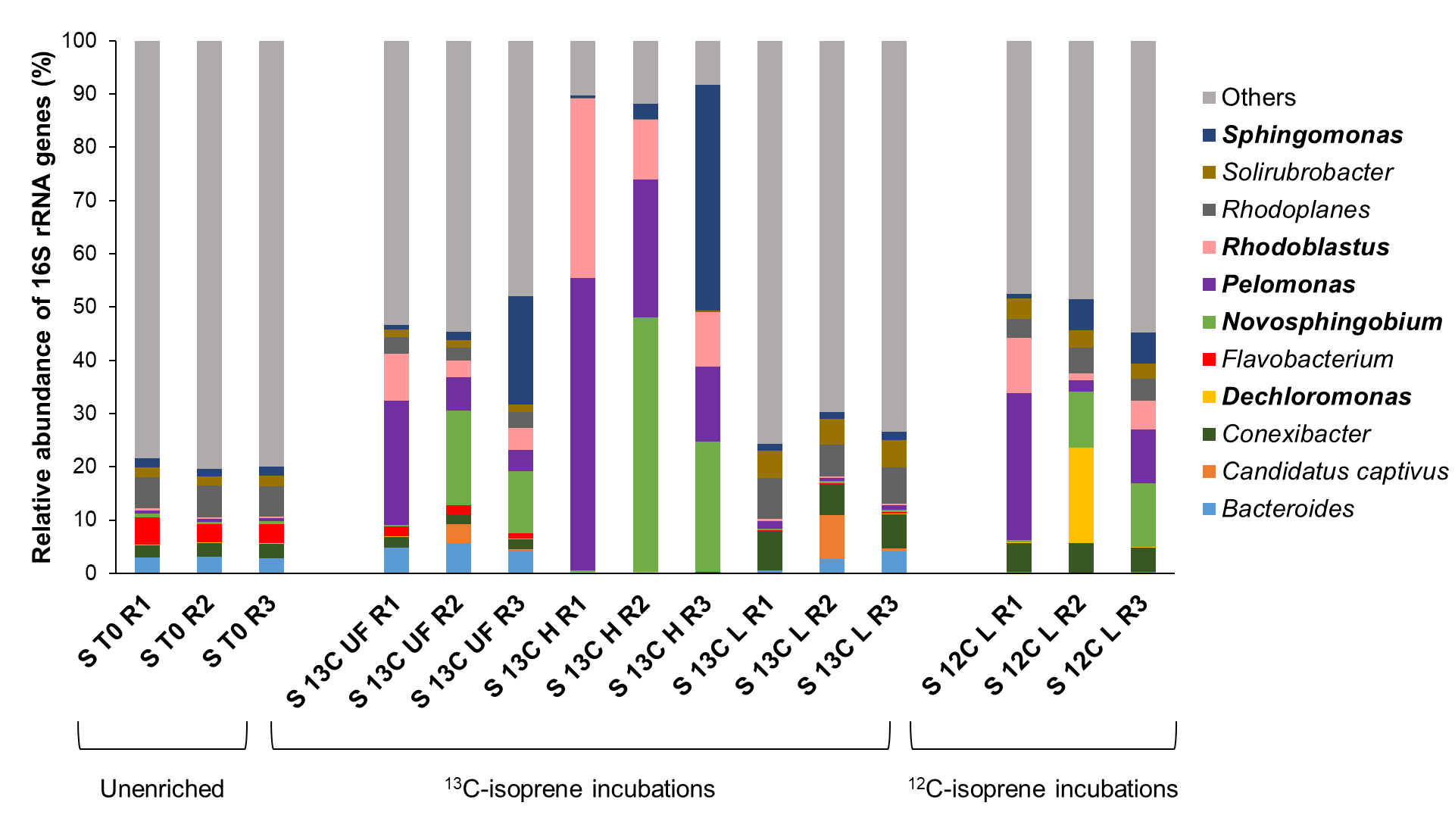


**Fig S2. Bacterial community profiles of oil palm soil samples analysed by 16S rRNA gene amplicon sequencing.** T0: unenriched samples; 13C UF: unfractionated DNA from ^13^C-isoprene enrichments; 13C H: heavy DNA from ^13^C-isoprene incubations; 13C L: light DNA from ^13^C-isoprene samples; 12C L: light DNA from ^12^C-isoprene control samples. Only OTUs with >5% relative abundance in at least one of the replicates are represented. Genera present at >10% in any sample are shown in bold. OTUs with <5% relative abundance are shown as “Others”.


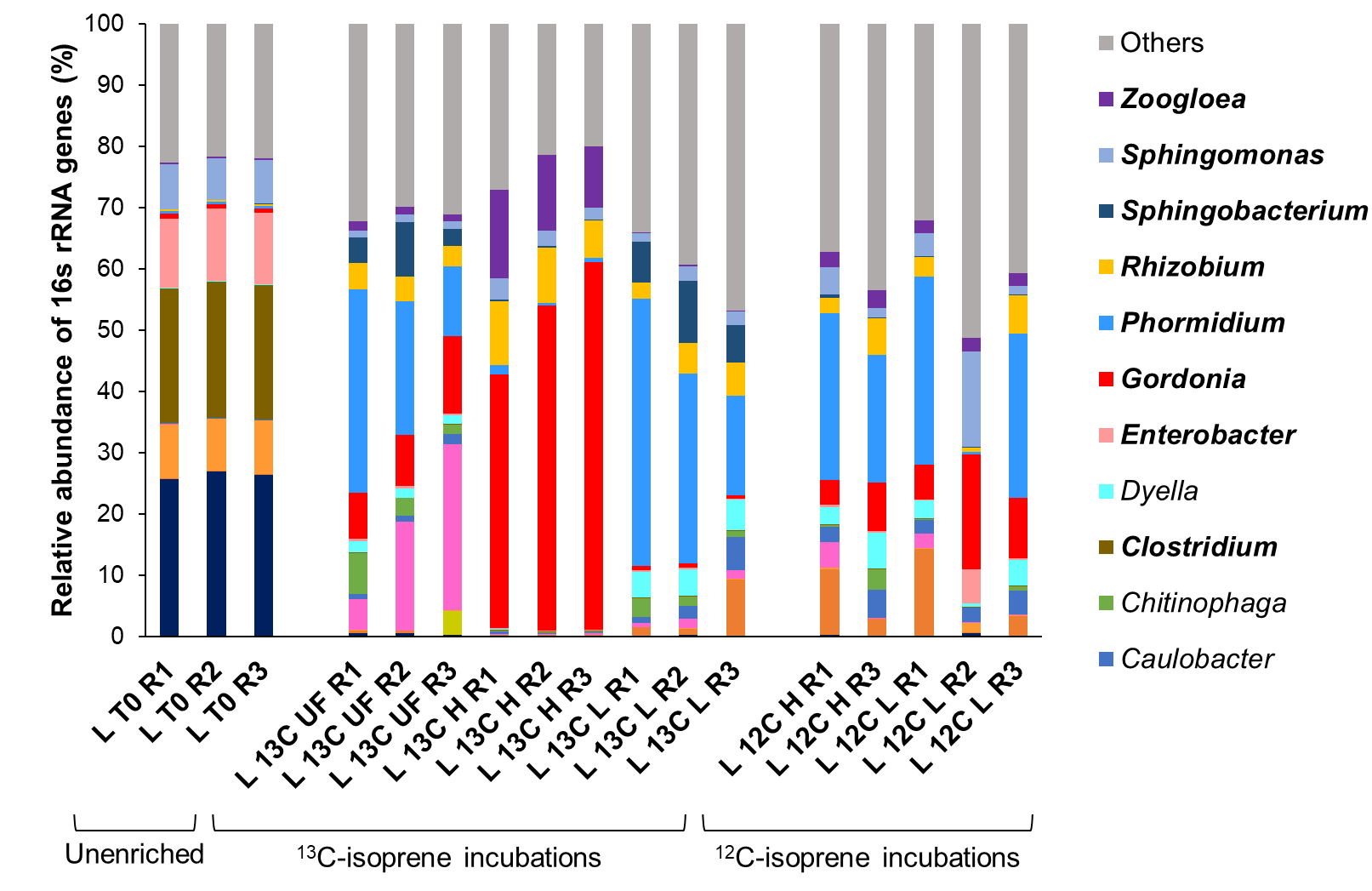


**Fig S3. Bacterial community composition of oil palm leaf samples analysed by 16S rRNA gene amplicon sequencing.** T0: unenriched samples; 13C UF: unfractionated DNA from ^13^C-isoprene enrichments; 13C H: heavy DNA from ^13^C-isoprene incubations; 13C L: light DNA from ^13^C-isoprene samples; 12C H: heavy DNA from ^12^C-isoprene control incubations; 12C L: light DNA from ^12^C-isoprene control samples. Only OTUs with >5% relative abundance in at least one of the replicates are represented. Genera present at >10% in any sample are shown in bold. OTUs with <5% relative abundance are recorded as “Others”.


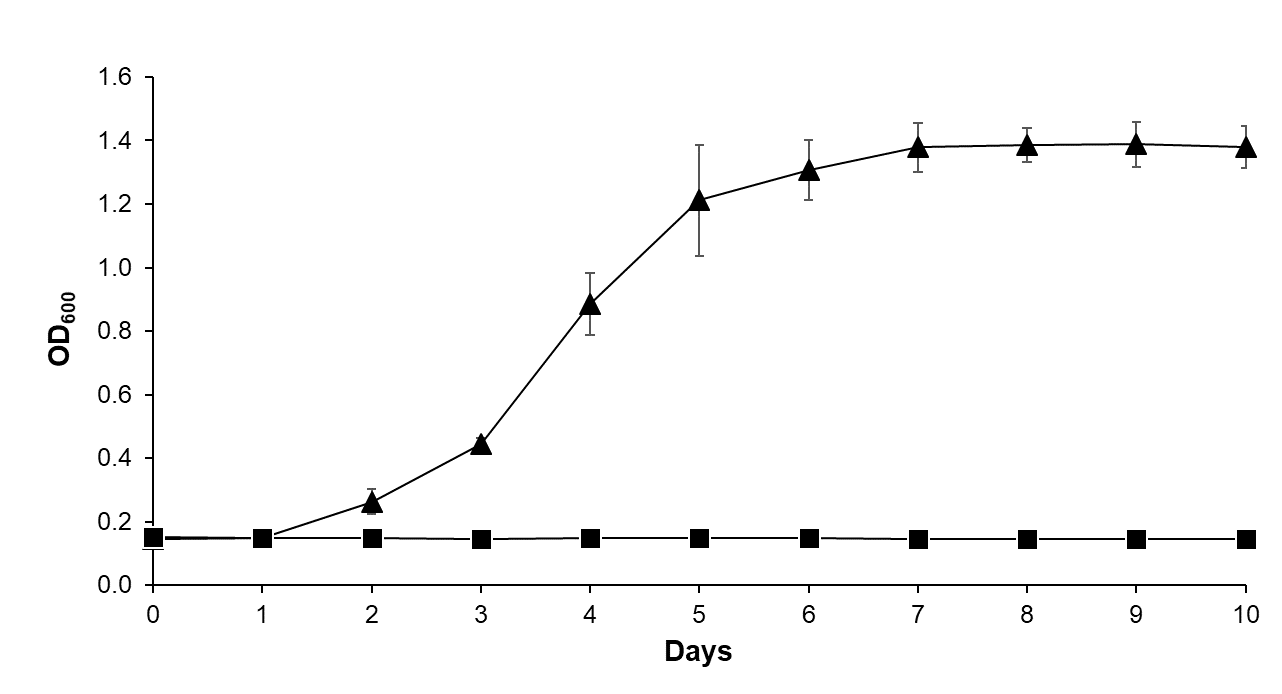


**Fig S4. Growth curve of *Variovorax* sp. OPL2.2 on isoprene as sole carbon and energy source.** Triangles: *Variovorax* sp. OPL2.2 cultures amended with isoprene (5%; v/v). Data points represent the average of three biological replicates with their respective standard deviations. Squares: *Variovorax* sp. OPL2.2 culture with no carbon source added (control; one replicate).


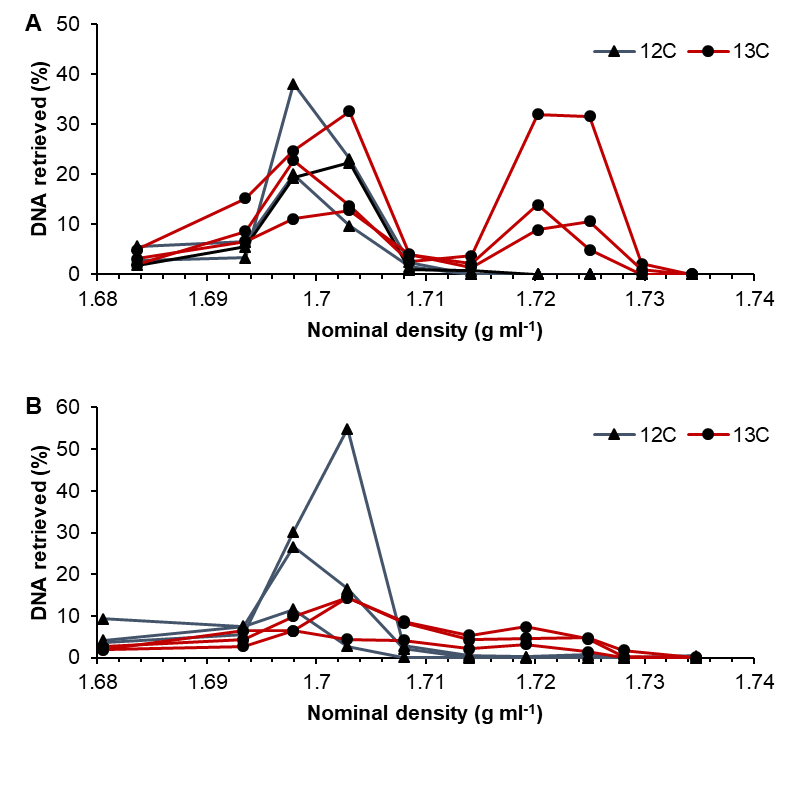


**Fig S5. DNA retrieved as function of density of each fraction recovered after isopycnic ultracentrifugation.** A: soil samples incubated with ^12^C (blue line) or ^13^C-isoprene (red line); B: leaf incubations with ^12^C (blue line) or ^13^C-isoprene (red line).


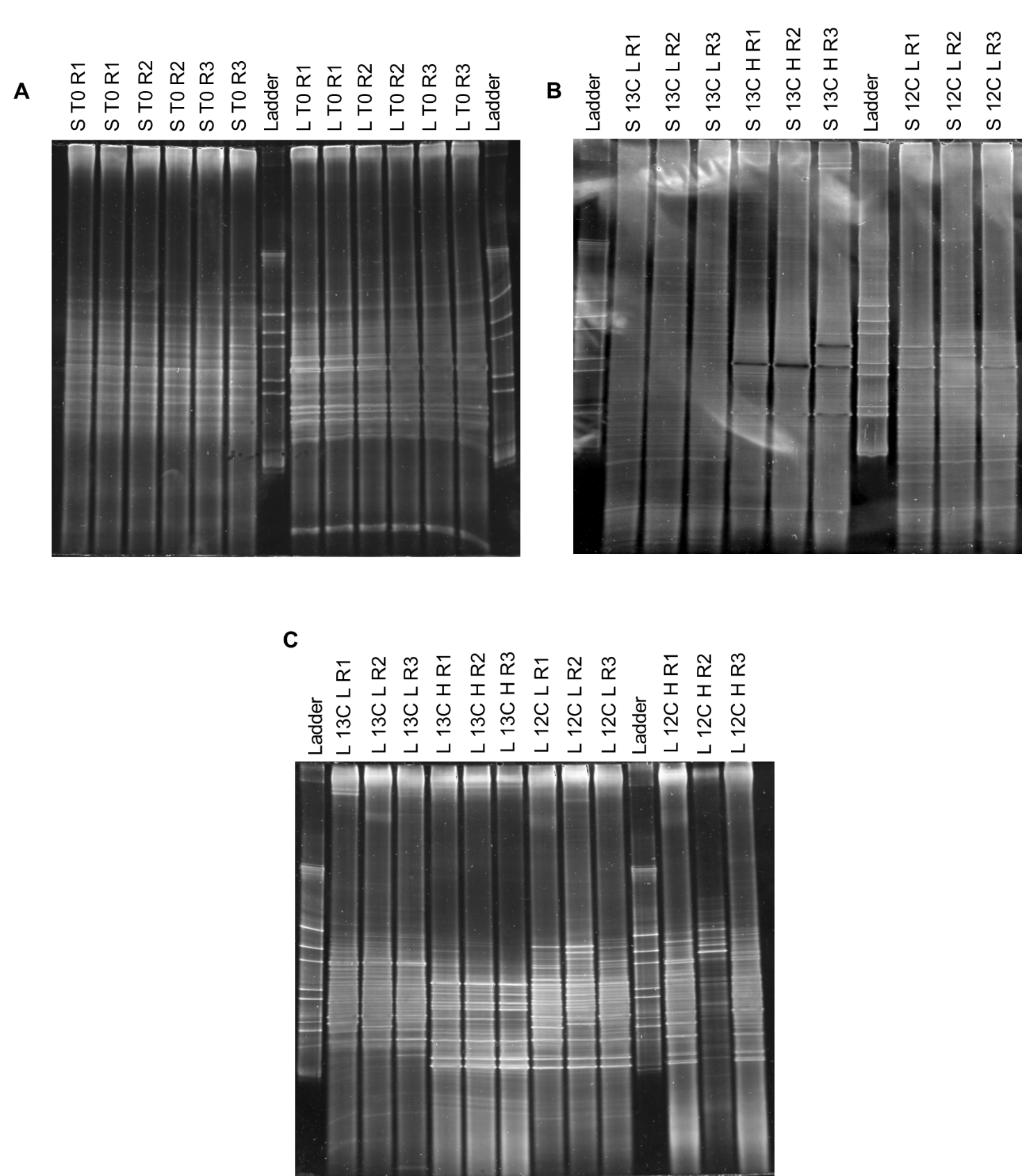


**Fig S6. 16S rRNA gene profiles of oil palm soil and phyllosphere samples analysed by DGGE.** A: biological replicates from unenriched soil (S T0) and phyllosphere (L T0) samples, each of which was run in duplicate. B: biological replicates from heavy (H) and light (L) soil incubations with ^13^C or ^12^C-isoprene (control). C: biological replicates from heavy (H) and light (L) leaf samples enriched ^13^C or ^12^C-isoprene.

**Table S1. Relative abundance of key isoprene-degrading bacterial genera in oil palm soil and leaf samples.**

| **Genus** | **S T0** | **L T0** | **S 13C H** | **L 13C H** |
| --- | --- | --- | --- | --- |
| *Gordonia* | 0.7 ± 0.2% | 0.8 ± 0.1% | 0.9 ± 0.1% | 51.4 ± 9.3% |
| *Novosphingobium* | 0.6 ± 0.1% | 2.8 ± 0.1% | 24.2 ± 23.8% | 0.3 ± 0.1% |
| *Pelomonas* | 0.5 ± <0.1% | 0.4 ± 0.1% | 31.7 ± 21.0% | 0.4 ± 0.1% |
| *Rhodoblastus* | 0.4 ± 0.1% | 0.2 ± <0.1% | 18.4 ± 13.3% | 0.3 ± 0.1% |
| *Sphingomonas* | 1.5 ± 0.1% | 7.1 ± 0.3% | 15.2 ± 23.6% | 2.6 ± 0.7% |
| *Zoogloea* | 0.2 ± <0.1% | 0.3 ± 0.1% | 0.3 ± 0.1% | 12.3 ± 2.2% |

Relative abundance of bacterial genera in oil palm incubations was analysed by 16S rRNA gene amplicon sequencing. Values shown represent the average of three biological replicates with their respective standard deviations. S T0: unenriched soil samples; L T0: unenriched leaf samples; S 13C H: heavy DNA from soil samples enriched with ^13^C-isoprene; L 13C H: heavy DNA from leaf samples enriched with ^13^C-isoprene.

**Table S2. Statistics for metagenome assemblies**.

|  | **S T0** | **S 13C H R1-2** | **S 13C H R3** | **L T0** | **L 13C H R1-3** |
| --- | --- | --- | --- | --- | --- |
| **Contigs** | 138,886 | 36,166 | 22,852 | 76,131 | 114,878 |
| **Largest contig (bp)** | 75,971 | 703,868 | 1,215,493 | 732,750 | 621,426 |
| **Total length (bp)** | 101,440,201 | 64,831,442 | 49,671,873 | 152,876,094 | 150,393,192 |
| **GC (%)** | 63.9 | 64.9 | 64.6 | 56.5 | 66 |
| **N50** | 684 | 2,852 | 5,677 | 5,018 | 1,565 |
| **N75** | 568 | 1,078 | 1,322 | 1,171 | 798 |
| **L50** | 50,076 | 3,502 | 959 | 3,953 | 18,953 |
| **L75** | 91,069 | 13,317 | 6,174 | 21,847 | 54,079 |
| **Bins** | 4 | 9 | 7 | 28 | 24 |

Metagenomes from unenriched soil (S T0) and leaf (L T0) samples and ^13^C-heavy fractions from soil (S 13C H R1-2 and S 13C H R3) and leaf (L 13C H R1-3) incubations were assembled with metaSPAdes using kmers 21, 33 and 55. Quality of the assemblies was analysed by MetaQUAST and bins obtained using MaxBin2.

**Table S3. Metagenome assembled-genomes (MAGs) that contain genes encoding proteins homologous to IsoABCDEF (E<1e-40).**

| **MAG** | **Metagenome**  **of origin** | **Size (Mbp)** | **N50** | **Completeness**  **(%)** | **Contamination**  **(%)** | **Strain heterogeneity (%)** |
| --- | --- | --- | --- | --- | --- | --- |
| *Novosphingobium* | S 13C H R3 | 3.7 | 441,005 | 99.5 | <0.1 | 0 |
| *Rhizobiales* | S 13C H R3 | 4.1 | 59,990 | 97.6 | 2.5 | 79 |
| *Gordonia polyisoprenivorans* | L 13C H R1-3 | 6.1 | 194,257 | 99.8 | 0.9 | 40 |
| *Zoogloeaceae* | L 13C H R1-3 | 5.2 | 49,423 | 98.7 | 2.1 | 10 |
| *Ralstonia* | L 13C H R1-3 | 4.5 | 5,039 | 79 | 6.4 | 0 |

MAGs were reconstructed from metagenomic sequencing of ^13^C-heavy DNA from soil (S 13C H) and leaf (L 13C H) incubations with ^13^C-isoprene. MAGs completeness and contamination was assessed and taxonomically assigned using CheckM (see Methods). N50 is calculated for contigs.

**Table S4. MAGs genes encoding polypeptides homologous to proteins involved in isoprene metabolism from ratified isoprene-degrading strains.**

***Novosphingobium* MAG**

| **Gene** | **Description** | **Closest protein from ratified isoprene degrader** | **Amino acid identity (%)** | **Coverage (%)** |
| --- | --- | --- | --- | --- |
| ***isoA*** | Hydroxylase α-subunit | IsoA from *Sphingopyxis* sp. OPL5 | 95.2 | 100 |
| ***isoB*** | Hydroxylase γ-subunit | IsoB from *Sphingopyxis* sp. OPL5 | 100 | 85.1 |
| ***isoC*** | Rieske-type ferredoxin | IsoC from *Sphingopyxis* sp. OPL5 | 100 | 78.6 |
| ***isoD*** | Coupling protein | IsoD from *Sphingopyxis* sp. OPL5 | 100 | 90.6 |
| ***isoE*** | Hydroxylase β-subunit | IsoE from *Sphingopyxis* sp. OPL5 | 83.7 | 100 |
| ***isoF*** | Flavoprotein NADH reductase | IsoF from *Sphingopyxis* sp. OPL5 | 76.2 | 100 |
| ***isoG*** | Racemase | IsoG from *Sphingopyxis* sp. OPL5 | 91.1 | 100 |
| ***isoH*** | Dehydrogenase | IsoH from *Sphingopyxis* sp. OPL5 | 89.4 | 100 |
| ***isoI*** | Glutathione-*S*-transferase | IsoI from *Sphingopyxis* sp. OPL5 | 89.8 | 100 |
| ***isoJ*** | Glutathione-*S*-transferase | IsoJ from *Sphingopyxis* sp. OPL5 | 90.6 | 98 |
| ***aldH1*** | Aldehyde dehydrogenase | AldH1 from *Sphingopyxis* sp. OPL5 | 85.3 | 100 |

***Rhizobiales* MAG**

| **Gene** | **Description** | **Closest protein from ratified isoprene degrader** | **Amino acid identity (%)** | **Coverage (%)** |
| --- | --- | --- | --- | --- |
| ***isoA*** | Hydroxylase α-subunit | IsoA from *Sphingopyxis* sp. OPL5 | 84.9 | 100 |
| ***isoB*** | Hydroxylase γ-subunit | IsoB from *Sphingopyxis* sp. OPL5 | 67.4 | 97 |
| ***isoC*** | Rieske-type ferredoxin | IsoC from *Sphingopyxis* sp. OPL5 | 61.2 | 92 |
| ***isoD*** | Coupling protein | IsoD from *Ramlibacter* sp. WS9 | 60 | 92 |
| ***isoE*** | Hydroxylase β-subunit | IsoE from *Sphingopyxis* sp. OPL5 | 54.8 | 98 |
| ***isoF*** | Flavoprotein NADH reductase | IsoF from *Sphingopyxis* sp. OPL5 | 54.9 | 99 |
| ***isoG*** | Racemase | IsoG from *Sphingopyxis* sp. OPL5 | 72.9 | 96 |
| ***isoH*** | Dehydrogenase | IsoH from *Sphingopyxis* sp. OPL5 | 74.3 | 100 |
| ***isoI*** | Glutathione-*S*-transferase | IsoI from *Sphingopyxis* sp. OPL5 | 72.9 | 95 |
| ***isoJ*** | Glutathione-*S*-transferase | IsoJ from *Sphingopyxis* sp. OPL5 | 69.9 | 100 |
| ***aldH1*** | Aldehyde dehydrogenase | AldH1 from *Sphingopyxis* sp. OPL5 | 64.7 | 99 |

***Gordonia polyisoprenovorans* MAG**

| **Gene** | **Description** | **Closest protein from ratified isoprene degrader** | **Amino acid identity (%)** | **Coverage (%)** |
| --- | --- | --- | --- | --- |
| ***isoA*** | Hydroxylase α-subunit | IsoA from *Gordonia polyisoprenovorans* i37 | 98.8 | 100 |
| ***isoB*** | Hydroxylase γ-subunit | IsoB from *Gordonia polyisoprenovorans* i37 | 82.1 | 100 |
| ***isoC*** | Rieske-type ferredoxin | IsoC from *Gordonia polyisoprenovorans* i37 | 99.1 | 100 |
| ***isoD*** | Coupling protein | IsoD from *Ramlibacter* sp. WS9 | 99 | 95 |
| ***isoE*** | Hydroxylase β-subunit | IsoE from *Gordonia polyisoprenovorans* i37 | 98.8 | 100 |
| ***isoF*** | Flavoprotein NADH reductase | IsoF from *Gordonia polyisoprenovorans* i37 | 99.4 | 100 |
| ***isoG*** | Racemase | IsoG from *Gordonia polyisoprenovorans* i37 | 98.3 | 100 |
| ***isoH*** | Dehydrogenase | IsoH from *Gordonia polyisoprenovorans* i37 | 99.1 | 100 |
| ***isoI*** | Glutathione-*S*-transferase | IsoI from *Gordonia polyisoprenovorans* i37 | 100 | 100 |
| ***isoJ*** | Glutathione-*S*-transferase | IsoJ from *Gordonia polyisoprenovorans* i37 | 94.7 | 100 |
| ***aldH1*** | Aldehyde dehydrogenase | AldH1 from *Gordonia polyisoprenovorans* i37 | 83 | 100 |
| ***aldH2*** | Aldehyde dehydrogenase | AldH2 from *Gordonia polyisoprenovorans* i37 | 94.8 | 100 |
| ***gshB*** | Glutathione synthetase | GshB from *Gordonia polyisoprenovorans* i37 | 99.1 | 98 |
| ***coADR*** | CoA disulfide reductase | CoADR from *Gordonia polyisoprenovorans* i37 | 94 | 100 |

***Zoogloeaceae* MAG**

| **Gene** | **Description** | **Closest protein from ratified isoprene degrader** | **Amino acid identity (%)** | **Coverage (%)** |
| --- | --- | --- | --- | --- |
| ***isoA*** | Hydroxylase α-subunit | IsoA from *Variovorax* sp. WS11 | 51.1 | 100 |
| ***isoB*** | Hydroxylase γ-subunit | IsoB from *Ramlibacter* sp. WS9 | 51.7 | 97 |
| ***isoC*** | Rieske-type ferredoxin | IsoC from *Ramlibacter* sp. WS9 | 51 | 93 |
| ***isoD*** | Coupling protein | IsoD from *Gordonia* sp. OPL2 | 44.9 | 85 |
| ***isoE*** | Hydroxylase β-subunit | IsoE from *Ramlibacter* sp. WS9 | 43 | 85 |
| ***isoF*** | Flavoprotein NADH reductase | IsoF from *Ramlibacter* sp. WS9 | 39 | 97 |

***Ralstonia* MAG**

| **Gene** | **Description** | **Closest protein from ratified isoprene degrader** | **Amino acid identity (%)** | **Coverage (%)** |
| --- | --- | --- | --- | --- |
| ***isoA*** | Hydroxylase α-subunit | IsoA from *Variovorax* sp. WS11 | 48.3 | 100 |
| ***isoB*** | Hydroxylase γ-subunit | IsoB from *Ramlibacter* sp. WS9 | 52.5 | 83 |
| ***isoC*** | Rieske-type ferredoxin | IsoC from *Ramlibacter* sp. WS9 | 47.6 | 92 |
| ***isoD*** | Coupling protein | IsoD from *Rhodococcus opacus* PD630 | 44.2 | 81 |
| ***isoE*** | Hydroxylase β-subunit | IsoE from *Nocardioides* sp. WS12 | 45.1 | 94 |
| ***isoF*** | Flavoprotein NADH reductase | IsoF from *Rhodococcus opacus* PD630 | 39.1 | 100 |

Homology of the polypeptides encoded by genes recovered from MAGs to proteins from ratified isoprene degraders was analysed by BLASTx (see Methods) and is expressed as amino acid identity.

**Table S5. ASVs retrieved from *isoA* amplicon sequencing analysis of ^13^C-heavy DNA from soil and leaf incubations.**

|  | **Closest IsoA sequence** | **Source** | **Amino acid  Identity (%)** | **Cover (%)** | **Soil Samples  RA (%)** | **Leaf Samples  RA (%)** |
| --- | --- | --- | --- | --- | --- | --- |
| ASV_1 | *Gordonia* *i37* | Isolate | 100 | 99 | 5.3±4.6 | 91.9±7.3 |
| ASV_2 | *Rhizobiales* | Metagenome | 100 | 99 | 35.5±6.1 | ND |
| ASV_4 | *Rhizobiales* | Metagenome | 92.5 | 99 | 6.1±5.3 | ND |
| ASV_5 | *Rhizobiales* | Metagenome | 100 | 99 | 8.9±8.7 | ND |
| ASV_7 | *Rhizobiales* | Metagenome | 100 | 99 | 4.2±3.7 | ND |
| ASV_8 | *Rhizobiales* | Metagenome | 71 | 99 | 2.2±3.8 | ND |
| ASV_9 | *Rhizobiales* | Metagenome | 98.7 | 99 | 1.8±3.1 | ND |
| ASV_11 | *Novosphingobium* | Metagenome | 100 | 99 | 3.7±3.4 | ND |
| ASV_12 | *Rhizobiales* | Metagenome | 98.9 | 99 | 1.4±1.3 | ND |
| ASV_13 | *Rhodococcus* AD45 | Isolate | 100 | 99 | 3.9±3.4 | 0.9±1.3 |
| ASV_14 | *Rhizobiales* | Metagenome | 98.1 | 99 | 1.8±0.4 | ND |
| ASV_15 | *Rhizobiales* | Metagenome | 96.2 | 99 | 2.5±2.4 | ND |
| ASV_16 | *Rhodococcus* AD45 | Isolate | 84.91 | 99 | 2.9±2.1 | ND |
| ASV_17 | *Rhizobiales* | Metagenome | 92.45 | 99 | 0.6±1 | ND |
| ASV_18 | *Rhizobiales* | Metagenome | 98.9 | 99 | 2.6±4.5 | ND |
| ASV_19 | *Rhizobiales* | Metagenome | 100 | 99 | 1.8±3.1 | ND |
| ASV_21 | *Rhizobiales* | Metagenome | 98.7 | 99 | 0.3±0.6 | ND |
| ASV_23 | *Rhodococcus* AD45 | Isolate | 99.37 | 99 | 1.2±1 | ND |
| ASV_24 | *Rhizobiales* | Metagenome | 100 | 99 | 0.2±0.3 | ND |
| ASV_26 | *Rhizobiales* | Metagenome | 98.1 | 99 | 0.2±0.4 | ND |
| ASV_29 | *Rhizobiales* | Metagenome | 100 | 99 | 1.8±3.2 | ND |
| ASV_30 | *Rhizobiales* | Metagenome | 98.7 | 99 | 0.1±0.3 | ND |
| ASV_34 | *Gordonia i37* | Isolate | 98.1 | 99 | ND | 3.4±5.6 |
| ASV_37 | *Rhizobiales* | Metagenome | 96.2 | 99 | 0.1±0.2 | ND |
| ASV_44 | *Novosphingobium* | Metagenome | 99.4 | 99 | 2.6±4.6 | ND |
| ASV_53 | *Rhizobiales* | Isolate | 99.4 | 95 | 0.5±0.8 | ND |
| ASV_59 | *Gordonia i37* | Isolate | 99.4 | 96 | ND | 1.8±3 |
| ASV_85 | *Gordonia* i37 | Isolate | 99.4 | 96 | ND | 2.7±3.8 |
| ASV_110 | *Rhizobiales* | Metagenome | 99.4 | 100 | 0.1±0.2 | ND |
| ASV_114 | *Gordonia* *i37* | Isolate | 100 | 99 | ND | 0.9±1.3 |
| ASV_122 | *Rhizobiales* | Metagenome | 95.6 | 100 | 0.1±0.2 | ND |
| ASV_160 | *Novosphingobium* | Metagenome | 100 | 99 | 0.2±0.4 | ND |
| ASV_168 | *Gordonia i37* | Isolate | 93.1 | 100 | ND | 1.7±3.0 |

Amino acid identity of ASVs retrieved from soil (S 13C H) and phyllosphere (L 13C H) samples to IsoA from ratified isoprene-degrading strains or MAGs reconstructed from metagenomes analysed in this study was determined by BLASTx (see Methods). Relative abundance (RA) of each ASV in heavy fractions from ^13^C-isoprene soil and leaf incubations represent the average of three biological replicates with their respective standard deviations. ND: not detected.

**Table S6. Location of oil palm trees used to set up soil and leaf DNA-SIP incubations.**

| **Sample** | **Latitude (N)** | **Longitude (E)** | **Elevation (m)** |
| --- | --- | --- | --- |
| Palong A | 2° 54' 03'' | 102° 39' 54'' | 55 |
| Palong B | 2° 54' 06'' | 102° 39' 33'' | 63 |
| Palong C | 2° 54' 03'' | 102° 38' 56'' | 33 |

Soil and leaf samples were collected from the same trees to allow comparison of the diversity of isoprene degraders from both environments.
